# The Treatment of Acquired Flat-Foot

**Published:** 1896-04-04

**Authors:** A. H. Tubby

**Affiliations:** Assistant Surgeon to, and in Charge of the Orthopædic Department at the Westminster Hospital, Surgeon to the National Orthopædic Hospital, and Surgeon to Out-patients, Evelina Hospital for Sick Children


### Medical Progress and Hospital Clinics.

[*The Editor will be glad to receive offers of co-operation and contributions from members of the profession. All letters should be addressed to* The Editor, at the Office, 428, Strand, London, W.C.]

### THE TREATMENT OF ACQUIRED FLAT-FOOT.

By A. H. Tubby, M.S.Lond., F.R.C.S.Eng., Assistant Surgeon to, and in Charge of the Orthopædic Department at the Westminster Hospital, Surgeon to the National Orthopædic Hospital, and Surgeon to Out-patients, Evelina Hospital for Sick Children.

(*Continued from page* 430.)

**Treatment of the Third Degree. Rigid Flat-foot.**—The causes of the rigidity are two, viz., the altered position of the bones, and the pain and tenderness incidental to this stage; which set up reflex contraction of the peronei and extensor longus digitorum. These muscles may be prematurely shortened or contractured, and the peronei tendons are sometimes dislocated forwards. But it is not always possible to be sure how much of the tightness of the peronei is reflex and how much is permanent. Before any division of these tendons is decided on it is well to take the patient completely off the feet for a fortnight. So much of the peroneal contraction may disappear that it is not necessary to perform tenotomy of those muscles. In the event of the peronei remaining contracted, after a few weeks' rest tenotomy should be done. In addition to those tendons the tendo Achilles may need division.

Cases of this degree may be treated in two ways, either by

(*a*) Forcible rectification under an anæsthetic and then retention of the foot in plaster.

(*b*) The gradual method by tenotomy, passive exercises, and the employment of a modified Scarpa's shoe.

Forcible rectification, either by the hand or Thomas's wrench. If the hand be employed (the method of Mr. Willett),[2] the patient must be fully under ether. The surgeon stands in front of the patient, and grasping the anterior part of the foot with both hands, with the thenar eminence of his right hand, if it be the patient's left foot, and the reverse if it be the right foot, pressing firmly on the head of the astragalus, steadily adducts and inverts the foot at the medio-tarsal and sub-astragaloid joints until the adhesions are felt to yield. Then rotation and circumduction movements are made at these joints, and finally the ankle joint is forcibly moved in all directions. The effects noticed are that the foot has come into better position at once, the peronei tendons are no longer tense, and the arch is partially or entirely restored. A thick flannel or cotton wool bandage is applied nearly to the knee, and then a plaster bandage put on from within outwards in figure of eight fashion. Anæsthesia is maintained until the plaster is dried, so that the foot remains fully adducted and inverted, and slightly flexed. Afterwards the patient may be allowed to walk if extra strips of plaster bandage are applied to the inner side and to the heel. The bandage should be worn for a month. If at the end of this time the foot is not in good position the process must be repeated. When the plaster is finally removed the patient is ready for exercise and a suitable walking instrument.

The same measures can be carried out with Thomas's wrench in place of the hand.

**Gradual Method.**—This has been the practice for some years past at the National Orthopædic Hospital. In the first place the peronei and extensor longus digitorum, and in some cases the Achilles tendon, are divided, and an external malleable iron splint put on until the punctures are healed. The affected foot is then placed in Mr. Adams's modification of Scarpa's shoe, designed for these cases, and the foot gradually adducted and rotated inwards. Due attention is also to be paid to the inversion at the ankle in the use of this apparatus. Care is taken that finally as much adduction as possible at the ankle and medio-tarsal joints is obtained. At least once a day, but better twice a day, the shoe is removed and passive exercises resorted to. The leg muscles are at the same time vigorously shampooed. In cases of this degree supports for walking are called for after this treatment. The double upright walking instrument is generally needed at first, and as the foot increases in strength the inner support may be removed, and still later the valgus boot, with pad and spring, substituted.

**Treatment of the Fourth Degree.**—It is in this degree and when wrenching under an anæsthetic has failed, that operative interference in the bones is alone justifiable. The operative measures are: (1) Resection of the astragalo-scaphoid joint (Ogston's operation); (2) Hare's[3] modification of Ogston's operation; (3) extirpation of the astragalus (Vogt's operation); (4) extirpation of the scaphoid (Golding-Bird and Davy); (5) transplantation of the posterior part of the os calcis (Gleich's operation); (6) excision of a wedge from the head and neck of the astragalus (Stokes' operation).[4]

**Ogston's Operation.**—An Esmarch's bandage is applied, and the foot having been rendered aseptic, is laid on its outer side. A horizontal incision one and a-half inch long is made down to the bone over the astragalo-scaphoid joint. The ligaments and periosteum are then dissected off the bones for about half an inch on either side of the incision, and the joint fully opened. With a chisel the cartilage and a thin layer of bone are removed from the astragalus, and similar structures from the scaphoid, in such a way as to leave on the latter bone a concave surface. The bones are then pegged together with ivory, the wound closed, and the foot put up in a plaster case. At the end of three months the patient is allowed to walk.

The result is an improved and, in a few cases, perfect arch, bony ankylosis and relief from pain. Walsham and Hughes state that they have only once had occasion to perform the operation themselves, and in this case the result was disappointing. I have not yet met with a case which could not be relieved by simpler measures.

Hare's modification of Ogston's operation consists in so treating the astragalus and scaphoid that the bones can be dove-tailed together.

**Stokes' Operation.**—An incision one and a-half inches in length is made below and parallel with the tendon of the tibialis posticus, over the most prominent part of the astragalus. A second incision joins this at right angles, and the small flaps thus made are turned back. From the neck and head of the astragalus a wedge of bone is taken away and the wound is closed and put up—according to the inventor of the operation—in a Dupuytren's splint. Plaster answers equally well.

It appears that Sir William Stokes' operation is the more scientific operation for the following reasons: (1) The medio-tarsal joint is left intact. (2) By prolonging the apex of the wedge well into the body of the astragalus, the centre of gravity at the "astragalus point"[5] is made to approximate to the inner side of von Meyer's triangle.

[2] St. Bartholomew's Hosp. Rep., Vol. XVIII., 1882. [3] Lancet, Nov. 9, 1895. [4] Trans. Acad. Med., Ireland, 1895, and Brit. Med. Jour., Dec. 1, 1894, p. 1,224, "Remarks on Flat-Foot." [5] This is the point which represents the centre of gravity of the astragalus, and is the highest spot in the axis of the trochlear surface.

